# Massive intra-abdominal irrigation fluid extravasation causing respiratory compromise and compressive hepatopathy during biportal lumbar foraminal endoscopic surgery: a case report

**DOI:** 10.1097/MS9.0000000000005299

**Published:** 2026-06-26

**Authors:** Subin Lim, Min-Seok Kang, Suk-Ha Lee, Tae-Hoon Kim

**Affiliations:** a Department of Orthopedic Surgery, Korea University Guro Hospital, Korea University College of Medicine, Seoul, Republic of Korea; b Department of Orthopedic Surgery, Konkuk University Medical Center, Konkuk University School of Medicine, Seoul, Republic of Korea

**Keywords:** biportal endoscopic, complications, hepatopathy, spine surgery

## Abstract

**Introduction:**

Biportal endoscopic spinal surgery is increasingly adopted as a minimally invasive technique with favorable clinical outcomes. Although neurologic complications, such as incidental durotomy and neural injury, are well-documented, serious visceral complications related to irrigation fluid extravasation are rarely reported. We describe a case of massive intra-abdominal irrigation fluid leakage following biportal extraforaminal lumbar discectomy, which resulted in acute respiratory failure and transient hepatic dysfunction.

**Methods:**

A 59-year-old male who developed sudden abdominal distension and dyspnea immediately after biportal endoscopic extraforaminal discectomy at L4–5 was evaluated. Clinical examination, computed tomography (CT), arterial blood gas analysis, and serial laboratory testing were performed. Radiologic attenuation values were assessed to differentiate simple fluid from hemorrhage. Percutaneous catheter drainage and diuretic therapy were administered for decompression and supportive management.

**Results:**

Physical examination revealed marked abdominal enlargement and tachypnea. CT demonstrated extensive retroperitoneal-to-intraperitoneal fluid accumulation with inferior vena cava compression and bilateral pleural effusions. Arterial blood gas analysis showed severe respiratory acidosis (pH 7.1). Laboratory findings indicated acute hepatic injury with markedly elevated alanine transaminases (peak ALT 1129 IU/L). Hounsfield unit measurements suggested simple irrigation fluid rather than hemorrhage. A large volume of intra-abdominal fluid was evacuated through percutaneous drainage and diuretic therapy, resulting in rapid clinical improvement. Liver enzyme levels normalized within 2 weeks, and the patient was discharged without residual symptoms.

**Conclusion:**

Massive irrigation fluid extravasation during biportal extraforaminal endoscopic lumbar surgery can precipitate abdominal compartment syndrome–spectrum physiology, leading to respiratory compromise and reversible compressive hepatopathy. Careful intraoperative monitoring of irrigation inflow–outflow balance and early recognition of intra-abdominal hypertension are essential to prevent life-threatening organ dysfunction.

## Background

Various minimally invasive techniques, including microscopic and endoscopic approaches, have been increasingly adopted in spinal surgery. Among them, biportal endoscopic spinal surgery has expanded its indications from the lumbar spine to the cervical region^[^1–3^]^. This technique offers several advantages, such as reduced soft tissue disruption, lower blood loss, and diminished postoperative pain, facilitating faster functional recovery^[^4–6^]^. However, as the use of endoscopic techniques continues to increase, a corresponding rise in procedure-related complications has been reported. The most commonly described adverse events include incidental durotomy and direct neural injury^[^7–10^]^. While these complications are relatively well recognized, irrigation-related complications remain uncommon and are less clearly characterized in the literature^[^11–13^]^.

HIGHLIGHTS

- To our knowledge, this is one of the very few documented cases of life-threatening intra-abdominal irrigation fluid extravasation during biportal endoscopic lumbar surgery, resulting in acute respiratory failure and reversible compressive hepatopathy. Although biportal endoscopic techniques are rapidly expanding worldwide, the literature has predominantly focused on neurologic or dural complications. Visceral compromise secondary to irrigation imbalance remains profoundly under-recognized and insufficiently characterized.
- Our report describes a 59-year-old patient who developed massive retroperitoneal-to-intraperitoneal fluid accumulation immediately after extraforaminal discectomy. The condition progressed to inferior vena cava compression, bilateral pleural effusions, severe respiratory acidosis (pH 7.1), and marked transaminase elevation (peak ALT 1129 IU/L), consistent with pressure-induced hepatic dysfunction. Prompt percutaneous decompression and diuretic therapy resulted in rapid physiological reversal and full recovery.
- This case is clinically significant for three reasons:
  1. It demonstrates that irrigation-related complications in biportal spine surgery can progress to abdominal compartment syndrome–spectrum organ failure.
  2. It provides radiological, physiological, and biochemical evidence supporting reversible compressive hepatopathy as a consequence of intra-abdominal hypertension.
  3. It highlights the critical importance of strict intraoperative irrigation inflow–outflow monitoring in dual-portal systems, where pressure imbalances may not be intuitively detected.
- As endoscopic spine surgery continues to expand in its indications and global adoption, failure to recognize this complication could result in catastrophic outcomes. We believe this report provides urgent and practice-changing clinical awareness for spine surgeons and perioperative teams.

Endoscopic spinal surgery requires continuous irrigation to maintain a clear operative field. However, pressurized irrigation fluid may infiltrate surrounding soft tissues and, in certain circumstances, track into adjacent anatomical compartments^[^8,9,14^]^. Excessive fluid extravasation can result in elevated intra-abdominal pressure and subsequent organ dysfunction. A similar phenomenon has been described in hip arthroscopy, where irrigation fluid leakage has led to abdominal compartment syndrome (ACS). In such cases, patients developed marked abdominal distension following arthroscopic procedures for femoroacetabular impingement. Because the primary intraoperative concern in hip arthroscopy typically focuses on neurovascular injury – such as damage to the pudendal nerve, lateral femoral cutaneous nerve, or circumflex femoral vessels – irrigation-related complications may not be immediately recognized^[^15,16^]^.

Thus, in biportal endoscopic spine surgery, the separation of inflow and outflow channels may make real-time irrigation balance less intuitive compared with single-portal systems. It is very important that the operating surgeon or assistants monitor both flows of irrigation fluid during surgeries. We report an unusual case of a patient with a life-threatening accumulation of massive intra-abdominal free fluid due to irrigation leakage during biportal endoscopic spinal surgery.

## Case presentation

**Figure 1.**
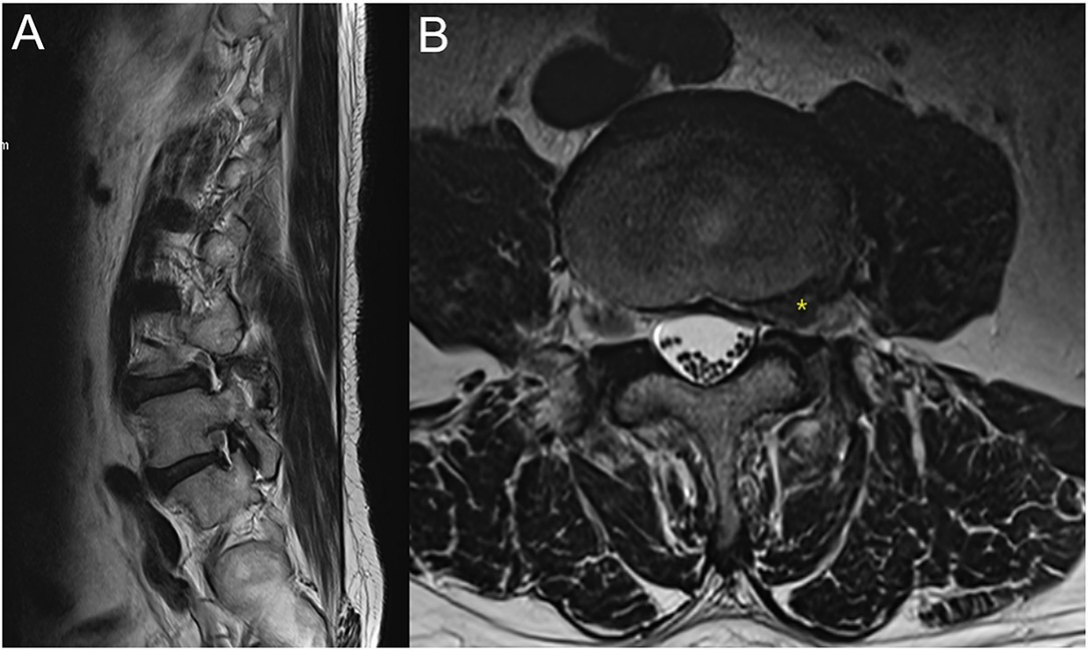
The preoperative magnetic resonance image (MRI) of the lumbar spine. (a) Sagittal; (b) axial cut on T2WI. The MRI showed disc extrusion (a yellow asterisk) in the left extra-foraminal zone with cranial migration, causing L4 nerve root compression.

A 59-year-old man with well-controlled hypertension and no previous trauma came to the emergency department of our hospital with the chief complaint of abdominal distension and dyspnea soon after spinal surgery. He reportedly underwent a biportal endoscopic discectomy (extra-foraminal approach) at the L4-L5 level at another orthopedic hospital. The preoperative magnetic resonance imaging (MRI) showed extra-foraminal disc extrusion causing L4 nerve root compression (Fig. 1).

In terms of his general appearance, the patient’s abdomen was seriously enlarged, with tenderness across the whole area. He suffered from tachypnea, with a respiratory rate of 34 per minute. A plain radiograph showed haziness in the left lung area, indicating pleural effusion, and severe distension with ileus in the abdomen.

**Figure 2.**
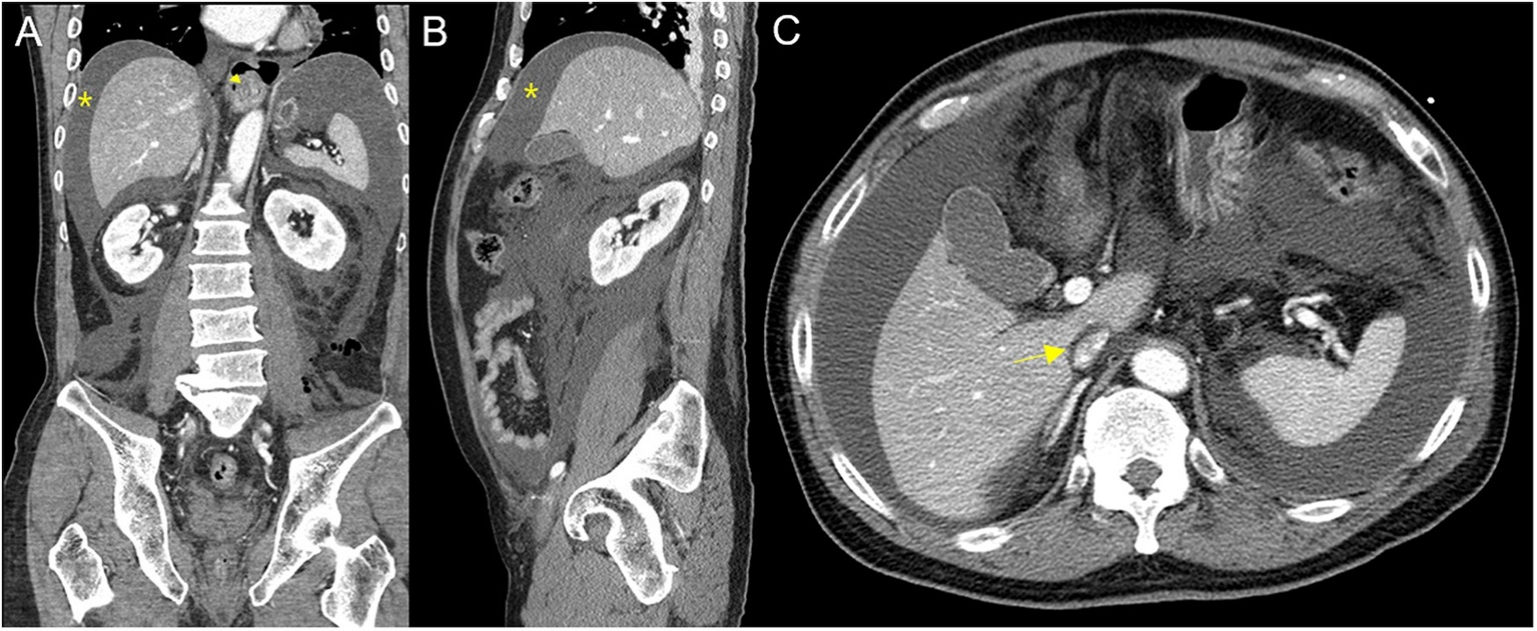
The computed tomography (CT) scan of the abdomen at the time in the emergency department. (a) Coronal; (b) sagittal; (c) axial cut. The coronal and sagittal cuts showed massive intra-abdominal fluid (yellow asterisk) infiltrating the left retroperitoneal space, compressing the kidney vertically and displacing the liver medially and posteriorly. In the coronal view, high intra-abdominal pressure caused the stomach to migrate upward, resulting in hiatal herniation (yellow arrowhead). The axial cut revealed a remarkable finding in the portal vein, which was narrowed by high intra-abdominal pressure (yellow arrows).

A CT scan was performed in the emergency department (Fig. 2). There was a large amount of collected fluid in the retroperitoneal and intraperitoneal spaces, with air bubbles in the former and in the left lower paraspinal muscles. In the right upper side of the intraperitoneum, the right upper margin of the liver was detached from its peritoneal membrane, where a massive amount of intraperitoneal fluid had accumulated. The Hounsfield unit of the abdominal CT was 8–12, implying simple fluid and not acute blood. Moreover, it showed a hiatal hernia induced by high intra-abdominal pressure. The axial view showed a large amount of fluid had collected in the intraperitoneal space, with remarkable signs that the inferior vena cava (IVC) had been narrowed and compressed by the high intra-abdominal pressure. Additionally, there was pleural effusion in both lungs, though it had collected more in the left lung. However, there was no focal lesion on the liver nor any abnormal signs on either kidney.

After laboratory tests and arterial blood gas analysis, respiratory acidosis was diagnosed with a pH of 7.1 (normal range: 7.35–7.45), 50.0 mmHg of partial pressure CO_2_ (normal range: 35-45 mmHg), and 75.0 mmHg of partial pressure O_2_ (normal range: 83–108 mmHg). There was a compensatory consumption of bicarbonate, indicated by a level of 6.20 mmol/L (normal range: 21-28 mmol/L), along with severely increased lactic acid at 6.20 mmol/L (normal range: 0.7-2.5 mmol/L). After the liver function test, hepatic insufficiency was diagnosed due to the increased aspartate transaminase (AST) at 204 IU/L (normal range: 8-38 IU/L), alanine transaminase (ALT) at 419 IU/L (normal range: 4-44 IU/L), and total bilirubin at 1.88 mg/dL (normal range: 0.2-1.2 mg/dL).

**Figure 3.**
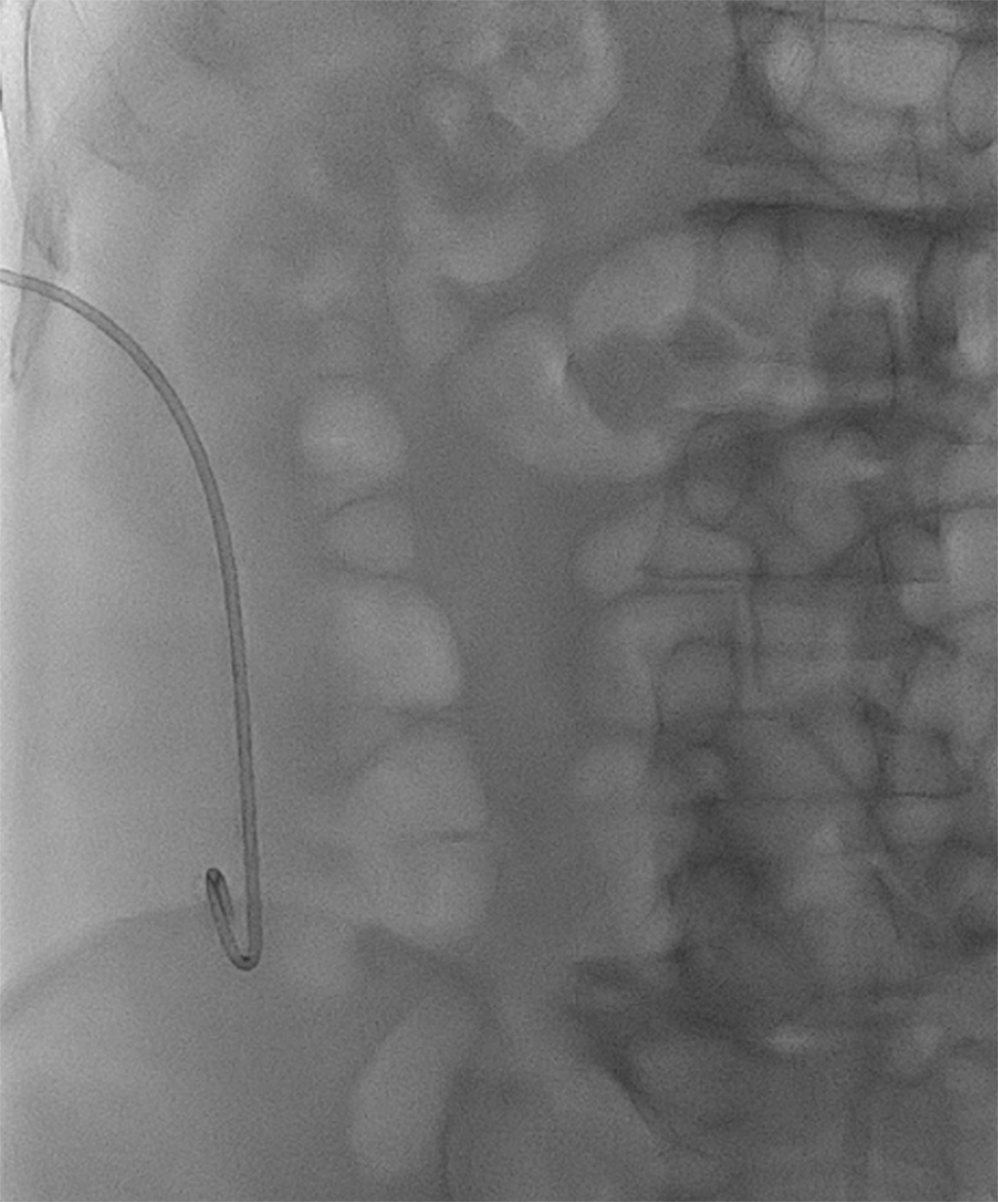
Percutaneous catheter drainage (PCD) was installed by a radiologist in the right side of the abdominal cavity. Over 3 days, a total of 1,180 ml of fluid was drained by PCD.

**Figure 4.**
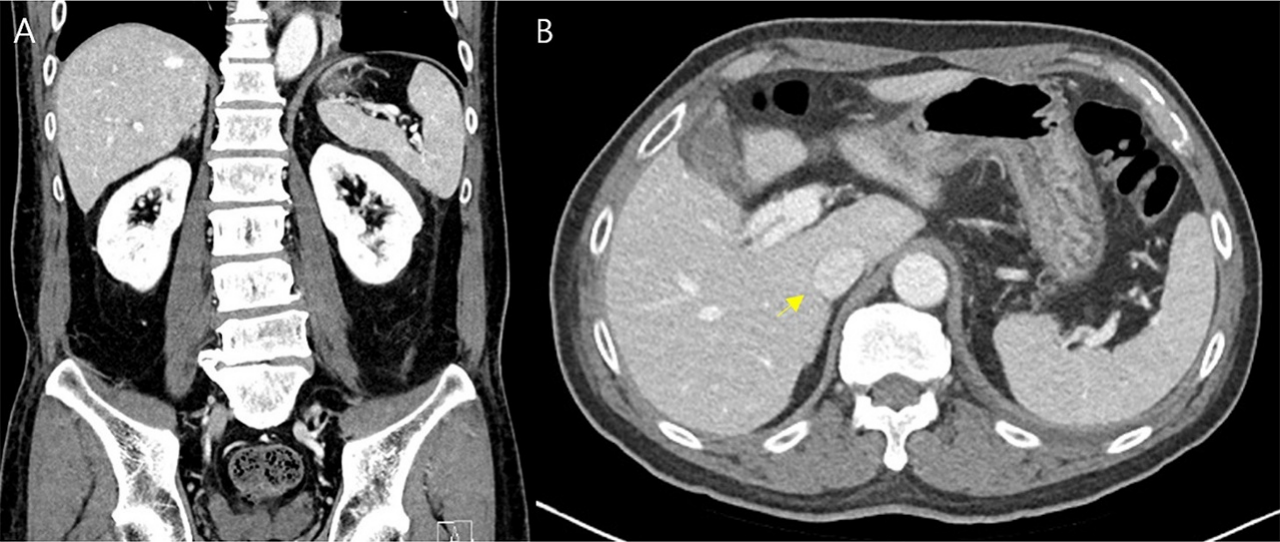
The CT scan taken on the 5th day of hospitalization. (a) Coronal; (b) axial cut. The CT showed that almost all of the fluid was drained from the abdominal cavity. In the coronal cut, the size of the internal organs had recovered, and the level of both diaphragms had descended to the normal level. It was a remarkable finding that the IVC had fully recovered its round shape from the narrowed shape in the axial cut (yellow arrow).

**Figure 5.**
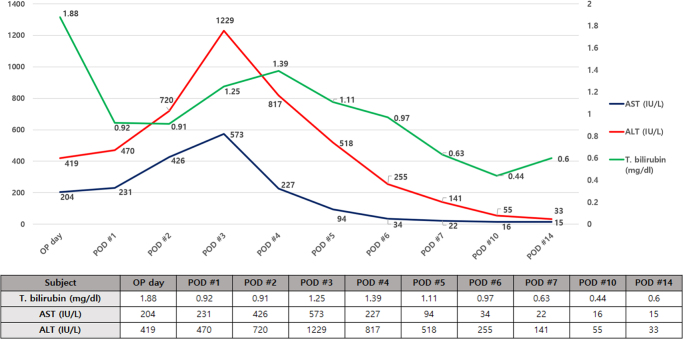
The timeline and line-graph of laboratory tests in serum aspartate transaminase (AST), serum alanine aminotransferase (ALT), and serum total bilirubin (T. Bilirubin). All tests showed higher values than the reference range on the day of surgery (OP day). The use of diuretics started at the same time as admission, along with Foley-catheterized urination, and intra-abdominal PCD) was installed the day after the operation (POD#1). The levels of AST and ALT peaked on postoperative day 3 (POD#3) but rapidly normalized thereafter. The patient was discharged with all laboratory tests normalized on POD#14.

Consequently, the patient was diagnosed with respiratory acidosis and hepatic insufficiency, with a large amount of fluid collected in the peritoneal space; thus, he was first admitted to the general surgery department. The day after admission, percutaneous catheter drainage was performed in the abdominal cavity by the department of radiology (Fig. 3). A total of 1180 ml of fluid was drained over the course of 3 days. Simultaneously, a large amount of fluid was drained via diuretics, with Foley catheter urination at an average volume of 6000 ml per day over the course of 4 days. After 5 days, a second abdominal CT scan was carried out, which showed that almost all of the fluid had been drained from the abdomen (Fig. 4). After 9 days, the patient had fully recovered from the dyspnea and showed no pleural effusion upon a plain radiograph. From admission to discharge, laboratory tests were serially carried out. His alanine aminotransferase (ALT), aspartate transaminase (AST), and total bilirubin levels were checked to evaluate his liver function (Fig. 5). The level of ALT peaked at 1129 IU/L on the third day after admission; then, it rapidly recovered to the normal range. Two weeks after admission, laboratory tests and radiology showed the patient had fully recovered. His symptoms of abdominal distension and dyspnea had diminished, and he was discharged.

## Discussion

We report a rare but potentially life-threatening complication of biportal endoscopic lumbar foraminal surgery: massive retroperitoneal-to-intraperitoneal irrigation fluid extravasation resulting in acute abdominal distension, respiratory failure, and transient hepatic dysfunction. CT demonstrated extensive fluid accumulation with IVC compression and pleural effusions, while arterial blood gas analysis revealed severe respiratory acidosis. Marked transaminase elevation rapidly normalized following decompression, supporting a reversible pressure-mediated injury. Although endoscopic spine surgery complications such as durotomy or neural injury are well documented, irrigation-related visceral compromise remains uncommon and is, therefore, easily underrecognized^[^7,11,13^]^.

The clinical presentation is best interpreted as a secondary ACS-like physiology induced by iatrogenic fluid overload. Elevated intra-abdominal pressure (IAP) can impair venous return, reduce hepatic perfusion, and compromise diaphragmatic excursion, leading to respiratory deterioration and organ dysfunction^[^17^]^. While direct IAP measurement was not performed, the constellation of imaging findings, multi-organ involvement, and rapid improvement after drainage strongly supports ACS-spectrum pathophysiology. Hepatic dysfunction in this case likely reflects compressive hepatopathy secondary to IVC compression and reduced venous outflow, rather than primary hepatic injury. The dramatic rise and subsequent normalization of AST/ALT following fluid evacuation further corroborate a reversible hemodynamic mechanism. Prior spinal endoscopy reports have predominantly focused on durotomy-related complications or neurologic sequelae, with very limited documentation of visceral organ compromise, highlighting the rarity of this presentation.

Anatomically and technically, extraforaminal or transforaminal biportal approaches may predispose patients to such events. The surgical corridor communicates with the paraspinal and posterior psoas planes, allowing pressurized irrigation fluid to track into the retroperitoneum and subsequently the peritoneal cavity when containment is insufficient. Moreover, the biportal system employs independent inflow and outflow channels; imbalance or partial obstruction of outflow can silently increase local pressure and promote extravasation. Unlike single-portal systems, real-time fluid equilibrium may be less intuitive, emphasizing the importance of active irrigation monitoring.

A comparable phenomenon is well recognized in hip arthroscopy, where irrigation fluid extravasation can lead to ACS and respiratory compromise. In spinal endoscopy, however, such cases are rarely reported, which may delay recognition. The rapid reversal observed after percutaneous catheter drainage and diuretic therapy in our patient mirrors the hip arthroscopy experience and underscores that timely decompression is highly effective.

From a practical standpoint, sudden postoperative abdominal distension, unexplained tachypnea or hypercapnia, metabolic acidosis, or unexpected pleural effusion should raise suspicion for irrigation-related intra-abdominal hypertension (IAH) or ACS. Prompt imaging and decompressive intervention are essential. Preventive strategies include limiting irrigation pressure and flow, ensuring continuous and unobstructed outflow, minimizing prolonged high-pressure exposure in extraforaminal corridors, and assigning explicit responsibility for monitoring irrigation balance during surgery.

A limitation of this report is the absence of direct intra-abdominal pressure measurement. Nevertheless, the radiologic, physiologic, and biochemical findings provide a coherent pathophysiologic explanation. In conclusion, massive intra-abdominal irrigation fluid extravasation during biportal extraforaminal endoscopic lumbar surgery can precipitate ACS-spectrum organ dysfunction, including respiratory failure and compressive hepatopathy. Vigilant irrigation management and early recognition are critical to preventing catastrophic outcomes.

## Data Availability

All data generated or analyzed during this study are included in this published article.

## Notes

Written informed consent for the publication of this case report and accompanying images was obtained from the patient.
